# Evaluation of Volume of News Reporting and Opioid-Related Deaths in the United States: Comparative Analysis Study of Geographic and Socioeconomic Differences

**DOI:** 10.2196/17693

**Published:** 2020-07-10

**Authors:** Yulin Hswen, Amanda Zhang, Clark Freifeld, John S Brownstein

**Affiliations:** 1 University of California San Francisco Department of Epidemiology and Biostatistics Bakar Computational Health Sciences Institute San Francisco, CA United States; 2 Computational Epidemiology Lab Harvard Medical School Boston, MA United States; 3 Harvard University Cambridge, MA United States; 4 Northeastern University Boston, MA United States; 5 Innovation Program Boston Children's Hospital Boston, MA United States

**Keywords:** opioid epidemic, news media, geographic, socioeconomic, addiction, overdose

## Abstract

**Background:**

News media coverage is a powerful influence on public attitude and government action. The digitization of news media covering the current opioid epidemic has changed the landscape of coverage and may have implications for how to effectively respond to the opioid crisis.

**Objective:**

This study aims to characterize the relationship between volume of online opioid news reporting and opioid-related deaths in the United States and how these measures differ across geographic and socioeconomic county-level factors.

**Methods:**

Online news reports from February 2018 to April 2019 on opioid-related events in the United States were extracted from Google News. News data were aggregated at the county level and compared against opioid-related death counts. Ordinary least squares regression was used to model opioid-related death rate and opioid news coverage with the inclusion of socioeconomic and geographic explanatory variables.

**Results:**

A total of 35,758 relevant news reports were collected representing 1789 counties. Regression analysis revealed that opioid-related death rate was positively associated with news reporting. However, opioid-related death rate and news reporting volume showed opposite correlations with educational attainment and rurality. When controlling for variation in death rate, counties in the Northeast were overrepresented by news coverage.

**Conclusions:**

Our results suggest that regional variation in the volume of opioid-related news reporting does not reflect regional variation in opioid-related death rate. Differences in the amount of media attention may influence perceptions of the severity of opioid epidemic. Future studies should investigate the influence of media reporting on public support and action on opioid issues.

## Introduction

Each day in the United States, more than 130 people die from opioid overdose and its death toll only continues to rise [1]. The unprecedented magnitude and growth of the opioid crisis have given it a prominent place in news reporting. As mass media is a powerful influence on public opinion, monitoring trends and differences in the volume of opioid-related news stories may offer important insights for addressing this crisis.

Analyzing the content of popular news coverage is an established method for studying the public discourse surrounding health and social issues and how this discourse may influence public attitudes [2-10]. Large bodies of research have demonstrated the agenda-setting function of news media [11,12]. By focusing coverage on some topics rather than on others, news media can influence which issues audiences perceive to be more important and in more need of response. For example, an analysis of over 25 years of General Social Survey data showed that greater rates of newspaper reading and television viewing were associated with higher likelihoods of public desire of funding to be directed to addiction [6]. In another study, a time-series analysis of changing public opinion about the importance of illegal drug use in the United States showed that public views were largely driven by news media describing drug abuse as a *crisis* [7].

Of particular interest is how differences in the volume of media attention may translate into differences in government policy, funding, and resource deployment, and thereby affect particular geographic regions and social groups. For example, a content analysis of 100 popular press articles revealed that differential media coverage of whites and blacks in the opioid epidemic of the 2000s may have led to different public responses and policy interventions [13]. Another study found evidence that biased media coverage directed policy to be disproportionately aimed at specific stereotypes of drug users [14]. In these ways, the uneven landscape of opioid media reporting may play a major role in the differences in public health outcomes of the current US opioid epidemic.

As the opioid crisis is a relatively new public health problem, previous research on media coverage of the opioid epidemic in the United States is limited. Borwein et al [15] found that print news media in North America portrayed the opioid analgesic oxycodone as a social problem, which coincided with the reduction in oxycodone prescriptions by doctors in Nova Scotia, Canada, but the authors were not able to determine a causal relationship. McGinty et al [9] studied how opioid analgesic use was depicted by the US print and television media from 1998 to 2012, revealing that in the United States, the issue was commonly framed as a criminal justice problem requiring legal solutions rather than a remediable public health issue. Lillian Seklir and Lori Dorfman [5] found contrarily that in media coverage in Northern California, opioid addiction is routinely presented as both a public health and a criminal justice issue, but public health advocates and medical practitioners are largely absent from the coverage.

In this study, we developed a platform called DrugMap.org, a novel multistream real-time surveillance system that aggregates online news reports on opioid-related events, to investigate geographic and socioeconomic differences in the volume of opioid-related news reporting and opioid-related deaths in the United States. Our study expands on current research in four important ways: (1) Our real-time data set allows us to study the most recent (2018-2019) reports to uncover relevant trends in this fast-evolving public health issue. (2) Our expanded data set includes online news sources, which are significant because 93% of US adults report reading news online [16]. (3) The scope of our study encompasses all but 4 US states (Montana, North Dakota, South Dakota, and Wyoming). And (4) we dig more deeply into news trends by associating them with epidemiological data and study how differences in socioeconomic status and geography may contribute to potential discrepancies between news media and the true nature of the opioid crisis.

## Methods

### DrugMap Data

#### News Reports Mentioning Opioids From DrugMap

News reports about opioids from February 2018 to April 2019 were collected from DrugMap.org, a multistream news archive platform we developed that captures news media data using automated scripts. Using the Google News API, DrugMap extracts all online news articles from Google News whose body text contained key opioids and opioid-related terms that were developed in consultation with medical toxicologists: *opioid, oxycontin, dilaudid, suboxone, oxycodone, morphine, methadone, buprenorphine, hydrocodone, heroin, fentanyl, naloxone, vicodin, percocet, actiq, duragesic, sublimaze, naltrexone, codeine, tylox, tramadol, tapentadol, ultram, percodan, robitussin, demerol, roxicet, avinza, butrans, dolophine, embeda, exalgo, nucynta, opana, kadian, oxymorphone, levorphanol, dihydrocodeine,* and *zohydro.* Although we included a detailed list of terms, this search list may not be fully comprehensive. We did not limit search topics to opioid drug overdose or deaths and included all opioid-related news, in line with previous drug news studies that modeled drug epidemics based on volume of all media related to the drug of interest [17].

Geographic coordinates (latitude and longitude) were assigned to each report using a previously established geolocation engine, CLIFF-CLAVIN, a text mining tool that parses news articles to retrieve place mentions [18]. The location where the reported opioid-related event occurred was determined using place names mentioned in the news report text. US county information was derived by inputting latitude and longitude into the census geocode Python library [19].

The number of opioid-related news reports retrieved from DrugMap was 92,124, spanning from February 7, 2018 to April 8, 2019. Duplicate online news reports, news reports with the same body text and URL, and articles in geographies outside the United States were excluded. News reports that reported a granularity larger than state level (eg, New England) were also removed from the analysis as these articles could not be defined to a specific county. After data cleaning, the total number of reports mentioning opioids was 35,758 and represented all 50 states and 1789 unique counties. The data covered 56.93% (1789/3142) of all US counties. Of the 1789 counties represented by the DrugMap data, 14.76% (464/3142) also contained Centers for Disease Control and Prevention (CDC) opioid-related death data, and this subset was used in the regression analysis. The 464 counties represent 46 states plus Washington DC, with the 4 states not included being Montana, North Dakota, South Dakota, and Wyoming. To account for variation in county population, adjusted news volume was defined as the number of news reports per population and was calculated by dividing total report count by county population times 100,000 total population.

#### County-Level Opioid-Related Deaths

Population-adjusted rates of opioid-related deaths in 2017 were obtained from the Multiple Cause of Death data set produced by the Division of Vital Statistics of the National Center for Health Statistics at the CDC, accessible through the WONDER portal of the CDC [1]. This CDC data set was compiled from death certificates for US residents, with each entry containing a single underlying cause of death and up to 20 additional contributing causes. Because death certificates for drug overdose are not complete in identifying drugs involved (in 2017, 12% of drug overdose death certificates did not provide information on specific drugs involved), we aimed to be broad in our inclusion criteria for opioid-related deaths, querying for all deaths that contained an opioid in the multiple-cause-of-death category rather than the underlying-cause-of-death category. Specifically, the criteria for inclusion were the following ICD-10 codes: opioids (T40.0, T40.1, T40.2, T40.3, T40.4, or T40.6); natural and semisynthetic opioids (T40.2); methadone (T40.3); synthetic opioids, other than methadone (T40.4); and heroin (T40.1). Death rates were given in units of deaths per 100,000 total population in county. Retail opioid prescriptions dispensed per 100 persons were collected from the CDC Opioid Prescribing Rate database [20].

#### County-Level Demographic and Socioeconomic Data

Demographic and socioeconomic variables were used to assess how media reporting and death rate differed in their relationship to external variables. Data on census region membership (Northeast, South, Midwest, and West), county population, high-school attainment in county, percent white in county, median income in county, and percent rurality in county were obtained from the US Census Bureau of Statistics [21].

#### Descriptive and Visual Analysis

To examine spatial trends, population-adjusted opioid news volume from DrugMap was visualized on a geographic map of the United States. To examine social and demographic trends, 2 heatmaps were created: one showing opioid news volume per 100,000 total population in county on the axes of rurality and educational attainment and another showing opioid-related death rates per 100,000 total population in county on the same axes. These maps were created in the following manner: first, we identified counties that contained both news data and death data. Of this subset of counties, we created 25 groups based on quintiles of percent rurality in county and high-school graduation rate in county. For each group, we computed the average opioid news rate and average opioid-related death rate by taking the mean of the individual rates of the counties in each group. This allowed us to visualize differences in associations with socioeconomic factors for opioid-related deaths versus opioid reporting.

### Statistical Analyses

Ordinary least squares regression was used to model county death rate and county report rate. A Poisson model was initially considered with population as an offset, but upon evaluation this model was rejected because of overdispersion, a violation of model assumptions. We then chose ordinary least squares after checking that the assumptions of normality of the residual terms were met. Counties with both DrugMap and CDC death data were used for this analysis. In Model 1, we modeled opioid-related death rate in county as a linear function of county geographic, demographic, and socioeconomic variables to identify area-level characteristics of greater opioid-related mortality. In Model 2, we used ordinary least squares regression to model news reporting rate in county as a linear function of county death rate and county variables to identify areas of higher volume of news reporting. A number of studies have described county characteristics associated with opioid use [22,23]. These include educational attainment, racial composition, physician supply, opioid prescribing rate, income, and rurality. These variables were included as controls in Model 1 and Model 2. All analyses were conducted with Python statsmodels [24].

## Results

### Descriptive and Visual

**Figure 1:**
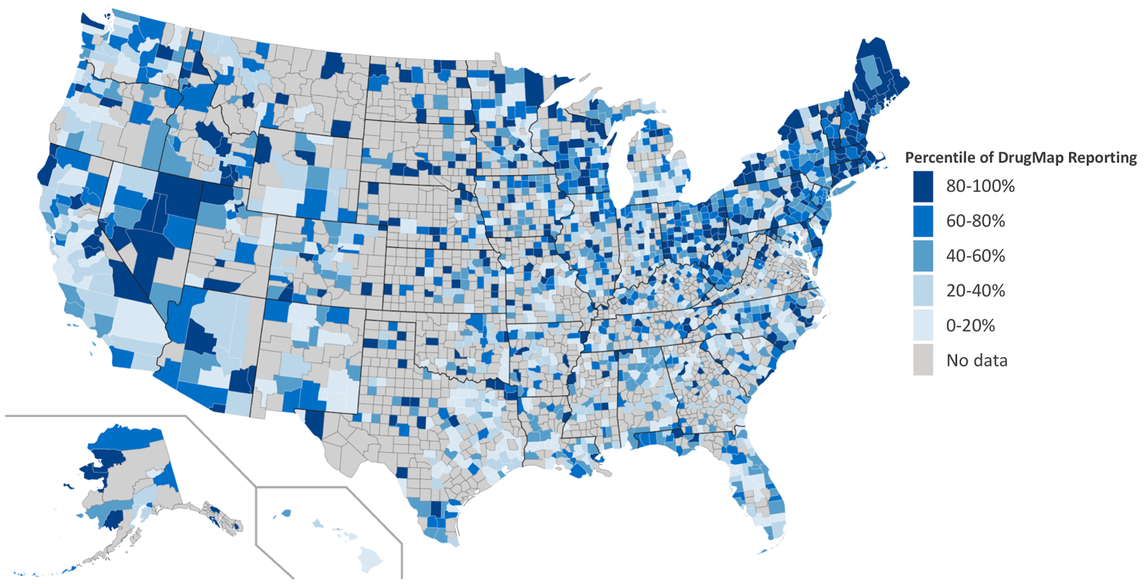
US Map of DrugMap reports in county divided by county population, 2018-2019.

The median number of reports per state was 565.5 (range 7-4169) and the median number of reports per county was 5 (range 1-1631). At the county level, the median was 9.35 reports per 100,000 total population (range 0.38-430.57 per 100,000 total population). Figure 1 displays the population-adjusted volume of DrugMap reporting across the United States by percentile category. As can be seen, opioid reporting volume is higher in the Northeast, especially in the areas of Maine, New Hampshire, Vermont, and Massachusetts. Other states with high opioid reporting relative to population size are Alaska and Nevada.

**Figure 2:**
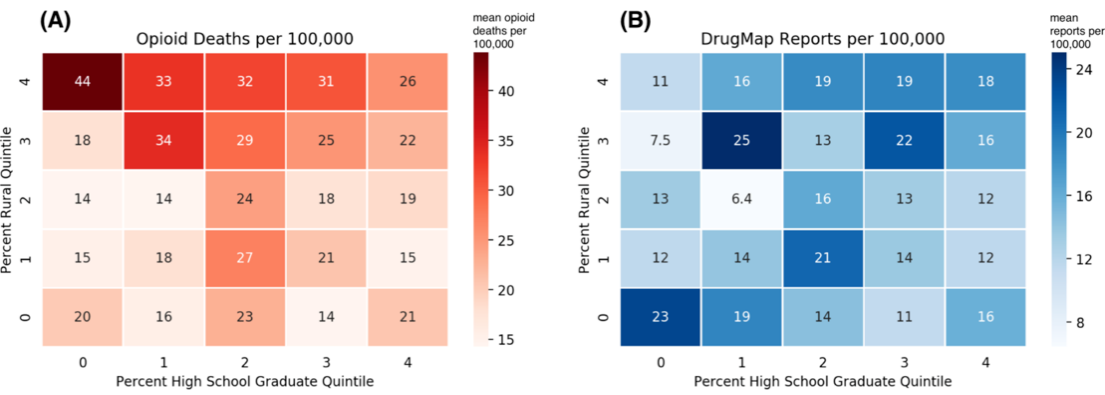
Heatmap showing distribution of opioid-related death rate (A) 2017 and DrugMap report rate (B) 2018-2019 by percent rural and percent high-school graduate.

Figure 2A shows the mean value of opioid-related death rate on the axes of percent rurality quintile and high-school graduation rate quintile, and Figure 2B shows the mean value of opioid reporting on the same axes. In the mortality heatmap, counties in higher quintiles of rurality and lower quintiles of education are shaded more darkly, indicating greater opioid-related death rates in rural and less educated populations. By contrast, the heatmap of DrugMap news reporting shows more variation and does not reflect the pattern observed for opioid-related death. When we compare the distribution of online opioid news from DrugMap with the US populations, we find that for rurality, the news data represented 53.7% of the first quintile (least rural), 15.3% of the second quintile, 4% of the third quintile, and 0.4% of the fourth/fifth quintile (most rural). For high-school graduation rate, the news data represented 2.6% of the first quintile (least educated), 8.5% of the second quintile, 18.2% of the third quintile, 23.9% of the fourth quintile, and 18.9% of the fifth quintile (most educated). These differences show that there exists greater news coverage about opioids relative to opioid mortality in urban and more educated areas.

### Regression

Model 1 regression results are presented in Table 1. For every percentage point increase in percent white at the county level, there was on average 28.02 less opioid-related deaths per 100,000 total population (*P*<.001), controlling for median income, rurality, high-school graduate rate, opioid prescribing rate, and geographic region. For every percentage point increase in percent rurality at the county level, there was on average 0.14 more opioid-related deaths per 100,000 total population (*P*<.01), controlling for all other variables. For every percentage point increase in percent high-school graduation rate at the county level, there was on average 0.86 less opioid-related deaths per 100,000 total population (*P*<.001), controlling for all other variables. Compared with the Midwest, the West had on average 9.99 less opioid-related deaths per 100,000 total population (*P*<.001), whereas there was no significant differences for the Northeast or South. Median income and opioid prescribing rate at the county level were not significantly associated with opioid-related death rate.

Model 2 regression results are presented in Table 1. Opioid mortality was positively associated with opioid news reporting; for every additional death per 100,000 total population at the county level, there was on average 0.40 more news reports per 100,000 total population (*P*<.001), controlling for median income, percent white, rurality, high-school graduate rate, opioid prescribing rate, and geographic region. For every dollar increase in median income at the county level, there was on average 0.0003 more news reports per 100,000 total population (*P*<.001), controlling for all other variables. For every percentage point increase in percent rurality at the county level, there was on average 0.17 less news reports per 100,000 total population (*P*<.01), controlling for all other variables. For every percentage point increase in percent high-school graduation rate at the county level, there was on average 0.65 more news reports per 100,000 total population (*P*<.01), controlling for all other variables. For every additional opioid prescription per 100 persons at the county level, there was on average 0.112 more news reports per 100,000 total population (*P*<.01), controlling for all other variables. Compared with the Midwest, the Northeast had 10.12 more news reports per 100,000 total population (*P*<.001), whereas the South and West exhibited no significant differences. Percent white at the county level was not significantly associated with news reporting frequency.

**Table 1:** Summary of regression results of Models 1 and 2^a,b^.

Dependent variable	Model 1				Model 2				
	Death rate				Report rate				
	Coefficient	Standard error	*P* value	95% CI		Coefficient	Standard error	*P* value	95% CI
Intercept	84.202	18.575	<.001	47.701 to 120.703		–24.040	19.152	.21	–61.678 to 13.598
Death rate	N/A^c^	N/A	N/A	N/A		0.397	0.047	<.001	0.305 to 0.489
Median income	–0.0001	6.28 × 10^–05^	.11	–0.000 to 2.32 × 10^–05^		–0.0003	6.37 × 10^–05^	<.001	–0.000 to –0.000
Percent white	28.019	6.431	<.001	15.382 to 40.656		0.410	6.652	.95	–12.662 to 13.483
Percent rural	0.143	0.054	<.01	0.036 to 0.249		–0.172	0.056	<.01	–0.282 to –0.061
Percent high-school graduate	–0.857	0.242	<.001	–1.332 to –0.381		0.649	0.248	<.01	0.162 to 1.135
Opioid prescribing rate	0.024	0.046	.55	–0.065 to 0.114		–0.112	2.438	.02	–0.202 to –0.022
Northeast	–0.786	2.054	.70	–4.823 to 3.251		10.115	2.078	<.001	6.031 to 14.200
South	–1.919	1.911	.32	–5.675 to 1.836		0.779	1.954	.69	–3.062 to 4.619
West	–9.991	2.418	<.001	–14.743 to –5.238		0.129	2.485	.96	–4.755 to 5.013

^a^Midwest was used as the reference group in both models.

^b^For both models, N=464.

^c^N/A: Not applicable.

## Discussion

Results from our study show that opioid-related mortality and opioid-related news reporting were positively correlated with each other: increases in opioid-related death rate at the county level were associated with increases in news reports. However, opioid-related mortality and opioid-related news reporting differed in how they related to geographic, demographic, and socioeconomic factors. Opioid-related deaths are less common in the geographic region of the West and more common in communities that are less educated, have a greater proportion of whites, and are more rural. While this finding regarding how opioid *mortality* relates to geography, education, and racial breakdown is consistent with prior studies [25], our analysis suggests that opioid-related *news reporting* follows different trends. Areas with greater news reporting tended to be more educated, more urbanized, and located in the Northeast. These discrepancies in news reporting rate and death rate, which can be interpreted as a sign of the magnitude of the opioid crisis in a certain area, highlight potential differences in the relative attention of news coverage across regions, rural areas, and socioeconomic status. More urban and educated areas receive greater media attention even though the opposite pattern is observed for opioid-related deaths. Previous research has shown that greater media attention and coverage increase public and governmental responses [6,13]. This increased attention may lead the public and the government to devote more resources and develop policy intervention targeted to areas that are less affected by the opioid epidemic.

In recent years, mass media has become increasingly relevant because its online transmission is immediate and its reach is broad. Because online news is often the first point of contact people have with current events, online sources prime people’s perceptions of the reported issues going forward and potentially override conflicting information received afterward [26]. For example, during the 1980s War on Drugs, media depiction of the cocaine epidemic as a “ghetto” problem is widely believed to have contributed to the public’s perception of African Americans and Latinos as addicts and criminals and white drug users as casualties and victims [13,27,28]. Another devastating case of differential media attention is the widely circulated news associating the measles vaccine with autism. News headlines prominently reported this association, and even though academics have long dispelled and discredited this link, this belief still persists because of the heightened media attention received by the original report and the lower media attention received by the studies that dismissed this association [29-31]. Through examining media trends for the current US opioid epidemic, we see that patterns of opioid media attention do not reflect the distribution of opioid-related deaths. This may lead to exacerbations of health disparities as previous studies have linked media attention with resource allocation. Lower media volume in areas with greater opioid use risk may reduce attention from government officials or public health agencies for these regions, leading to an increase in disparities. For instance, opioid-related deaths occur most in rural areas with lower education, suggesting that these areas are being affected the most. However, our results indicate that there may be less opioid-related news reporting and coming from these areas. This relative lack of news discussion could signal less awareness, interest, and investment in opioid issues in regions that are at greater risk.

### Limitations

Results from this study were obtained at the area level based on county variables, so conclusions must be drawn at the ecological and not at the individual level. First, issues of collinearity across these demographic and socioeconomic variables prevented us from including all socioeconomic and demographic variables into the model. The specific variables selected in this study were based on previous research on opioid-related events. Second, our regression models provide information on associations and cannot be deemed causal. Third, while we adjusted news volume to consider differences in county population, the measures of baseline news output and readership may have been more direct control variables; however, these were unavailable during the study. Using these measures may have yielded different results.

There are benefits and drawbacks to our methodology of using opioid-related deaths as opposed to opioid-related overdose deaths for our analysis. Although the variable of opioid overdose deaths is more specific and perhaps more directly interpretable, overdose death data from the CDC are limited in important ways. Specifically, CDC data on drug overdose deaths are derived from information provided on death certificates, and not all death certificates for overdose include information on drugs involved.

We chose not to specify drug overdose as the underlying cause of death for opioid-related mortality in order to encompass a broader view of the impact of the opioid epidemic across the United States. Although our data are still subject to the aforementioned data limitations, the broader scope of our inclusion criteria allows us to achieve a more comprehensive analysis. These limitations in national databases are reflected in our analysis as counties with poorer reporting, which tend to also be more rural, and are thus excluded. However, even with greater representation in more urban areas, our results demonstrate a relative lack of media opioid reporting in rural and less educated areas, thereby supporting the existence of biases in media reporting on opioids.

Finally, our study did not examine the language or content of opioid-related news reporting, such as positive and negative connotation or types of vocabulary used. Textual examination of media content should be conducted in follow-up studies as previous research has shown that framing can impact public perceptions and judgments toward certain socioeconomic groups [8,12,13,32]. Furthermore, while our study supports the existence of differences in reporting volume, it does not measure the effect of such trends on government support and action on opioid issues. Future studies should be carried out to empirically investigate the relationship between variation in news reporting volume and variation in realized efforts.

### Conclusion

Our study brings to light potential differences in the volume of media portrayal of the current opioid epidemic. Previous research and theory have demonstrated a strong link between media attention and its impact on public perception, future resource allocation, and population-level opioid use behaviors. Although our study did not directly test these consequences, our results provide evidence supporting the existence of these media disparities across the United States. Given the importance of news media in shaping public attitudes and government efforts, it is critical that news reporting on opioids is distributed equitably and accurately to prevent misperceptions. Future studies should investigate trends in the content of news reporting on the opioid epidemic and how framing of news stories may be affecting government support and action on opioid issues.
